# Neurometabolic and electrophysiological changes during cortical spreading depolarization: multimodal approach based on a lactate-glucose dual microbiosensor arrays

**DOI:** 10.1038/s41598-017-07119-6

**Published:** 2017-07-28

**Authors:** Cátia F. Lourenço, Ana Ledo, Greg A. Gerhardt, João Laranjinha, Rui M. Barbosa

**Affiliations:** 1 Center for Neuroscience and Cell Biology, University of Coimbra, Coimbra, Portugal; 2 Center for Microelectrode Technology, University of Kentucky, Lexington, USA; 3 Faculty of Pharmacy, University of Coimbra, Coimbra, Portugal

## Abstract

Spreading depolarization (SD) is a slow propagating wave of strong depolarization of neural cells, implicated in several neuropathological conditions. The breakdown of brain homeostasis promotes significant hemodynamic and metabolic alterations, which impacts on neuronal function. In this work we aimed to develop an innovative multimodal approach, encompassing metabolic, electric and hemodynamic measurements, tailored but not limited to study SD. This was based on a novel dual-biosensor based on microelectrode arrays designed to simultaneously monitor lactate and glucose fluctuations and ongoing neuronal activity with high spatial and temporal resolution. *In vitro* evaluation of dual lactate-glucose microbiosensor revealed an extended linear range, high sensitivity and selectivity, fast response time and low oxygen-, temperature- and pH- dependencies. In anesthetized rats, we measured with the same array a significant drop in glucose concentration matched to a rise in lactate and concurrently with pronounced changes in the spectral profile of LFP-related currents during episodes of mechanically-evoked SD. This occurred along with the stereotypical hemodynamic response of the SD wave. Overall, this multimodal approach successfully demonstrates the capability to monitor metabolic alterations and ongoing electrical activity, thus contributing to a better understanding of the metabolic changes occurring in the brain following SD.

## Introduction

Spreading depolarization (SD) is a slow propagating wave of massive but short-term depolarization of neuronal and glial cells, implicated in a wide spectrum of neuropathological conditions such as traumatic brain injury (TBI), subarachnoid hemorrhage, stroke, epilepsy and migraine aura^1–3^. It is triggered when a strong stimulus simultaneously depolarizes a minimum critical volume of brain tissue causing a drop in neuronal transmembrane resistance. The re-establishment of ionic gradients after SD, via activation of ATP-dependent pumps, is extremely demanding energetically. Coherently, SD is characterized by marked metabolic changes associated with increased ATP consumption accompanied by hemodynamic changes which are required to deliver metabolic substrates imposed by the increase in metabolic demand^4^.

In numerous neuropathological conditions, such as TBI, it is recognized that SD does not occur as an epiphenomenon, but can elicit further neuronal injury after the primary insult thus often worsening the outcome. In this regard, major relevance has been attributed to the imbalance of metabolic and vascular mechanisms required for the restoration of brain homeostasis. It is now well accepted that SD events elicit a significant decrease in extracellular glucose concentration alongside with an increase in lactate^5–9^. Importantly, the magnitude and profile of this metabolic disturbance impacts on neuronal viability and on the clinical outcome, as substantiated by the observation that persistent low glucose levels^10^ and increased lactate/glucose ratio^11^ are associated to unfavorable outcome in TBI patients. Thus, the understanding of the dynamics fluctuation of these metabolic substrates is of paramount importance for prognostication and definition of therapeutic strategies in the clinical setting^12^.

Our knowledge of brain metabolism has been significantly advanced by the ability to monitor neurometabolic events *in vivo* with high spatial, temporal and chemical resolution. Relevant information has been obtained by non-invasive neuroimaging techniques (*e.g*. MRI, PET and NMR) which allow us to monitor neurometabolic events without tissue disruption. While these approaches present great advantages with relation to clinical translation, they are still limited in terms of spatial and temporal resolution. Invasive approaches, such as rapid sampling microdialysis (rsMD) have also been valuable tools for monitoring metabolic changes associated with SD events both in animal models of brain injury and patients^6–8, 13^. However, the minute-to-minute time resolution limits the detection of rapid changes in the metabolic substrates. Few studies have successfully addressed neurometabolic events during SD with the higher temporal resolution (second-by-second) as that afforded by amperometric biosensors^5, 8^. In fact, real-time electrochemical measurements of neurochemicals in the brain extracellular space is frequently a challenging task due to the complexity of the chemical environment^14^.

Microelectrode arrays (MEAs) are a very attractive microelectrode platform for developing amperometric enzyme-based biosensors^15^. The well-defined and highly reproducible geometrical configuration of the recording sites in the MEAs is important to accomplish reproducible measurements of the targeted analytes with spatial resolution. Another advantage of MEAs is that they can be configured for multi-analyte detection^16, 17^. This is of major significance in cases where correlation of the dynamic changes of the analytes is intrinsically relevant, as is the case for lactate and glucose^18^.

In addition to the high temporal and spatial resolution, MEA-based biosensors offer another advantage as, once coupled to amperometry, they can concurrently provide local field potential (LFP)-related information. Using MEAs-based biosensors, Zhang and collaborators demonstrated that the high frequency component of the amperometric recordings is qualitatively and quantitatively correlated with LFPs under several experimental manipulations^19^. This approach, further validated by others^20, 21^, allows the correlation between neurochemical alterations and ongoing electrical activity with an unique spatial and temporal precision. The measurement of neuronal activity has been proven to be of utmost relevance to detect neurological dysfunctions in the clinical setting following TBI^22^. In particular, the spectral profile of the electrical events assessed by electroencephalography (EEG) and electrocortiography (EcoG) have been suggested to provide relevant information about SD, including its prediction^22–24^.

In the present study we aimed to develop an innovative multimodal approach, encompassing metabolic, electric and hemodynamic measurements, tailored to study SD events on basis of a high temporal and spatial resolution window. However, we anticipate the potential of this approach to other applications, for instance to elucidate the mechanisms underlying neurometabolic coupling. We used platinum multisite MEAs to design and develop a new dual lactate-glucose microbiosensor, which was extensively characterized to address the suitability for *in vivo* measurements.

We established a multimodal approach using a new ceramic MEA-based design directly implanted in the brain tissue, providing simultaneous neurometabolic and electrophysiological information. In addition, we monitored cortical cerebral blood flow by laser Doppler Flowmetry. Using this approach we successfully measured local rapid fluctuations in lactate and glucose associated with neuronal activity (LFP-related currents) in the cortex of anesthetized rats during SD.

## Results

### *In vitro* dual biosensor characterization

**Figure 1:**
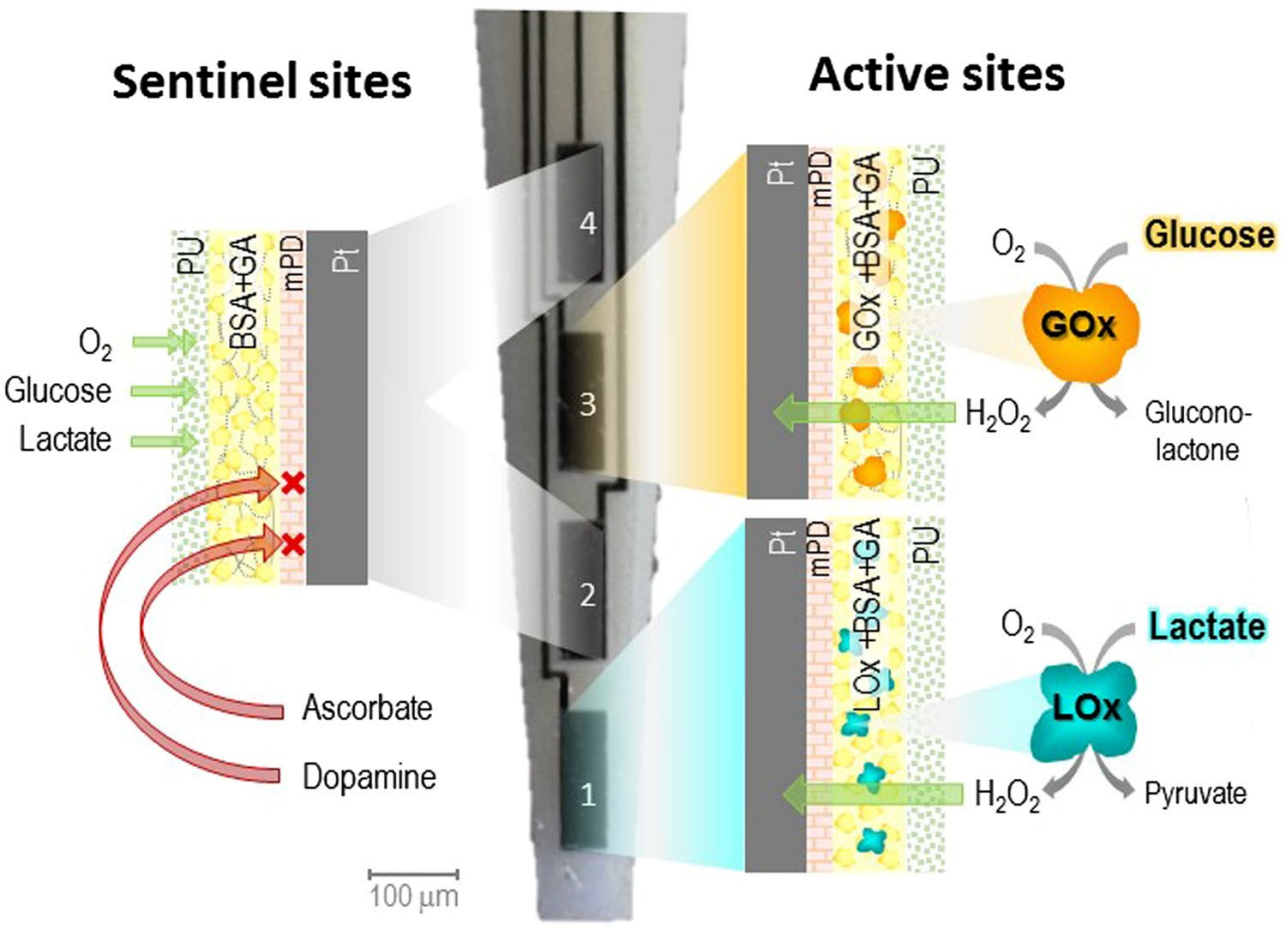
Schematic representation of the dual lactate-glucose biosensor developed from ceramic-based multisite microelectrode arrays (MEA) (125 µm thick) containing 4 platinum recording sites in line (R1, 50 × 150 µm^2^, spacing 50 µm). Sites 1 and 3 sites (active sites) were coated with a cocktail solution containing Lactate Oxidase (LOx) or Glucose Oxidase (GOx), BSA and glutaraldehyde (GA). Sites 2 and 4 (sentinel sites) were coated with the inactive protein matrix. The sites were further modified with an exclusion layer of *meta*-phenylenediamine (*m*-PD), to improve selectivity, and a diffusional barrier of polyurethane (PU), to extend the linear range for the substrate detection.

**Figure 2:**
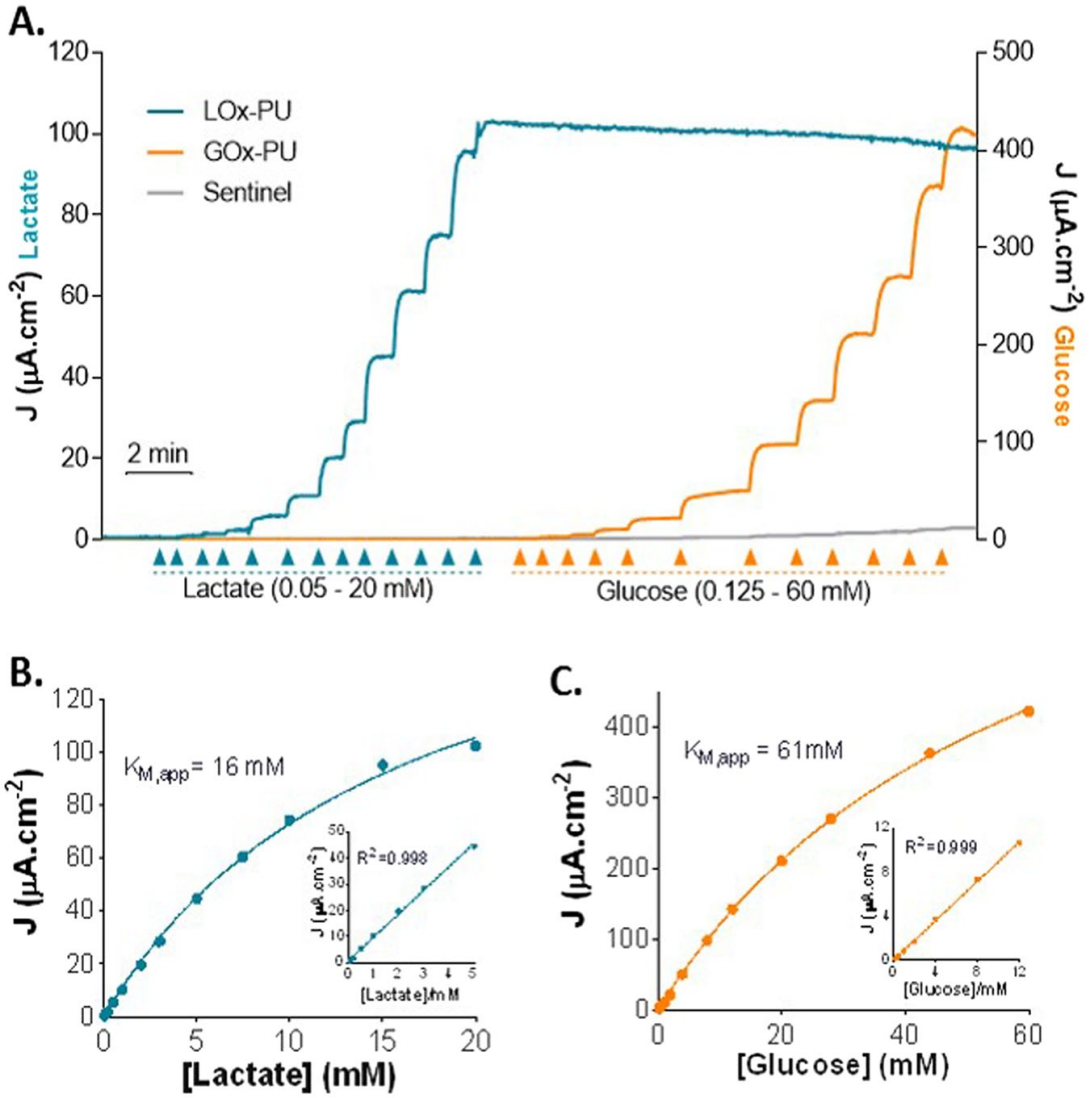
Enzyme kinetics of LOx-PU coated sites and GOx-PU coated sites towards lactate (blue line) and glucose (orange line) detection. Grey line represents the response of the sentinel site. (**A**) Representative calibration showing responses of LOx-GOx MBA to successive additions of lactate and glucose. The final concentrations after each addition was 0.05, 0.1, 0.2, 0.5, 1, 2, 3, 5, 7.5, 10, 15 and 20 mM for lactate and 0.125, 0.5, 1, 2, 4, 8,12, 20, 28, 44 and 60 mM for glucose. (**B**,**C**) Calibration plot of the average steady-state current as a function of lactate (**B**) and glucose (**C**) concentration. Data were fitted to the Michaelis–Menten equation by using non-linear regression analysis. Inserts: Linear regression of the response of the LOx-PU coated sites (**B**) and GOx-PU coated sites (**C**) to lactate and glucose, respectively.

Ceramic-based MEAs (R1 configuration) with 4 in-line Pt sites were configured for simultaneous detection of glucose and lactate by individually coating two of the sites with Lactate Oxidase (LOx) and Glucose Oxidase (GOx) (Fig. 1). Figure 2A shows a representative recording of the response of the LOx-GOx microbiosensor array (referred hereinafter as LOx-GOx MBA) to successive additions of increasing concentrations of lactate and glucose. The LOx- and GOx-coated sites exhibited a significant and selective response to lactate and glucose, respectively, while no significant current changes were detected at the sentinel sites. LOx-GOx MBAs with crosstalk between sites (>2%) were discarded. The response to both substrates followed Michaelis-Menten kinetics with a range of linearity (R^2^ > 0.99) up to 5 and 12 mM for lactate and glucose, respectively (Fig. 2B,C). The most relevant kinetics and analytical parameters are summarized in Table 1.

**Table 1:** Kinetic and analytical parameters of the LOx-GOx MBA response to lactate (LOx-PU sites) and glucose (GOx-PU sites).

	LOx-PU (n = 9)	GOx-PU (n = 7)
K_M, app_ (mM)	22 ± 6	80 ± 14
J_max_ (µA.cm^−2^)	55 ± 18	515 ± 170
Sensitivity (µA.cm^−2^.mM^−1^)	3.0 ± 1.0	10.7 ± 4.1
LOD (µM)	61 ± 19	41 ± 17
Response time (s)	10.6 ± 2.2	8.2 ± 1.4

**Figure 3:**
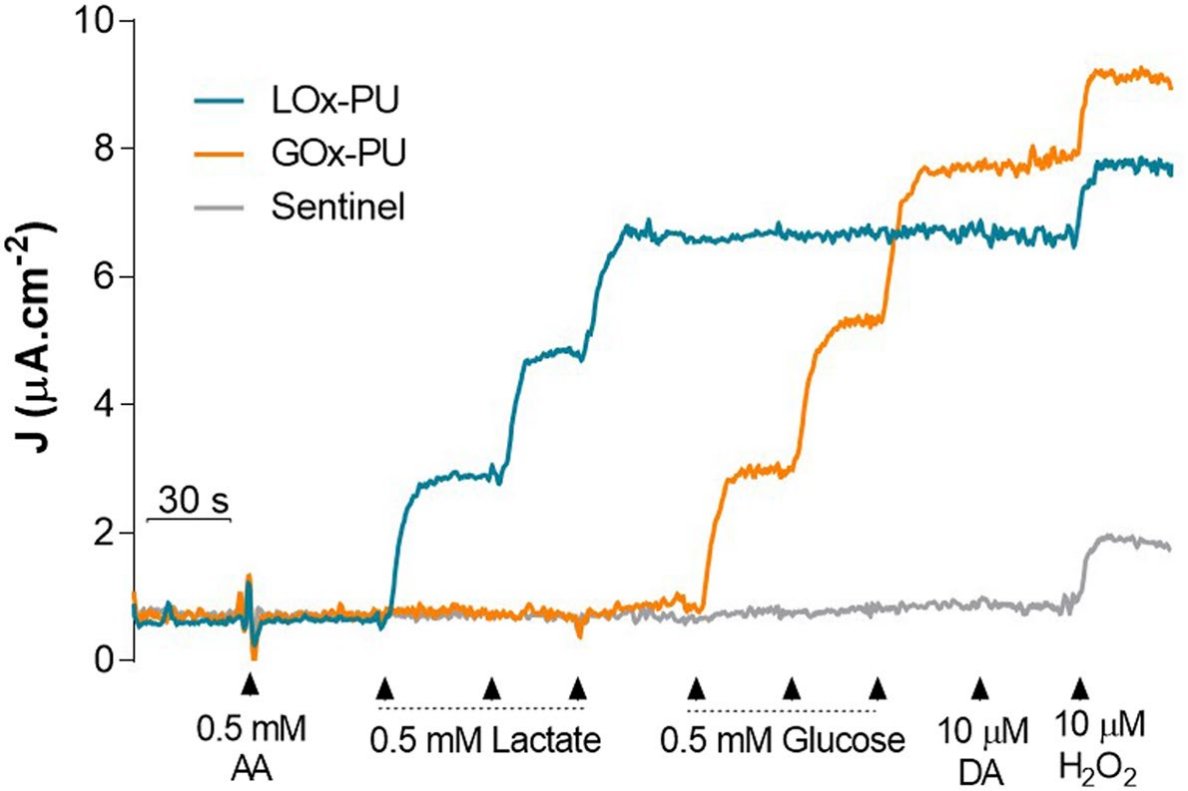
Representative calibration of the LOx-GOx MBA to lactate and glucose (0.5 mM) in the presence of interferents (0.5 mM of ascorbate and 10 µM of dopamine). H_2_O_2_ was used as test substance (positive control for sentinel sites).

To minimize the interference of undesirable electroactive compounds, the LOx-GOx MBA sites were modified with a poly-[*m*-phenylenediamine] exclusion layer^25^. In spite of the specificity of LOx and GOx for the corresponding substrates, the presence of electroactive compounds in the brain extracellular space limits biosensor specificity, namely ascorbate which extracellular concentration range of 200–400 μM^26–28^. A typical response of the LOx-GOx MBA to lactate and glucose in the presence of high concentrations of ascorbate (0.5 mM) is depicted in Fig. 3. The response to ascorbate was negligible in active and sentinel sites when compared to the response to lactate (0.5 mM) and glucose (0.5 mM) in active sites. The selectivity ratio (substrate:ascorbate) was 52 ± 11: 1 and 98 ± 28: 1 (n = 4) and the corresponding blocking efficiency 98% and 99% for LOx-PU and GOx-PU coated sites, respectively. No measurable current changes were detected by addition of 10 µM of dopamine while all sites responded to H_2_O_2_ (10 µM), used as a test molecule. The reported selectivity can be improved by taking advantage of the “self-referencing” recording technique through the off-line subtraction of the current recorded at the sentinel sites from that of the active sites^29^.

**Figure 4:**
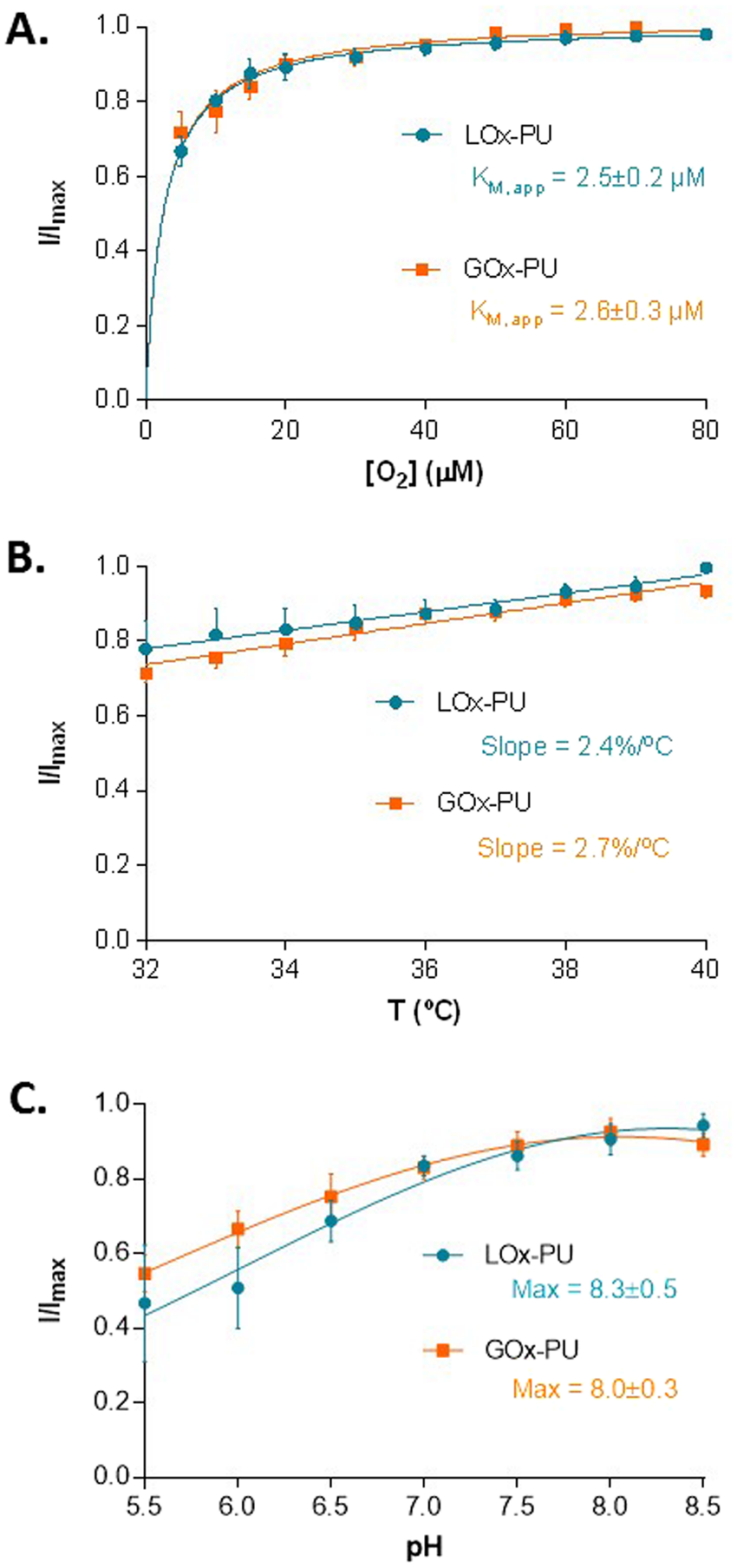
Evaluation of oxygen-, temperature- and pH- dependencies of the LOx-GOx MBA. (**A**) Average normalized current detected by LOx-PU and GOx-PU coated sites, respectively, to 1 mM lactate and 1 mM glucose as a function of oxygen concentration. Data were fitted to the Michaelis–Menten equation by using non-linear regression analysis. (**B**) Effect of temperature variation on the recorded current of LOx-PU and GOx-PU coated sites in the presence of 1 mM of lactate and glucose, respectively. (**C**) Effect of pH on the response of LOx-PU and GOx-PU coated sites to 1 mM of lactate and glucose, respectively. The response towards glucose was fitted to a Gaussian function.

To further address the suitability of LOx-GOx MBA to accurately measure lactate and glucose in the brain extracellular space we evaluated the dependencies of the LOx-GOx MBA for O_2_, pH and temperature (Fig. 4). As expected, considering that O_2_ is a co-substrate for both LOx and GOx, the response to lactate and glucose (1 mM) was affected by O_2_ concentration. The average steady-state current response for substrates plotted as a function of O_2_ concentration followed a Michaelis-Menten kinetics model and the K_M_(O_2_) calculated from the fits averaged 2.5 ± 0.2 µM and 2.6 ± 0.3 µM for lactate and glucose, respectively (Fig. 4A). Furthermore, we observed that the LOx-GOx MBA response to lactate and glucose was also dependent on temperature and pH albeit that variations were relatively low and similar for both analytes: 2–3%/°C and 1–2%/pH unit from 6.5 to 7.5 (Fig. 4B,C).

### *In vivo* validation of lactate and glucose measurements by the dual biosensor

**Figure 5:**
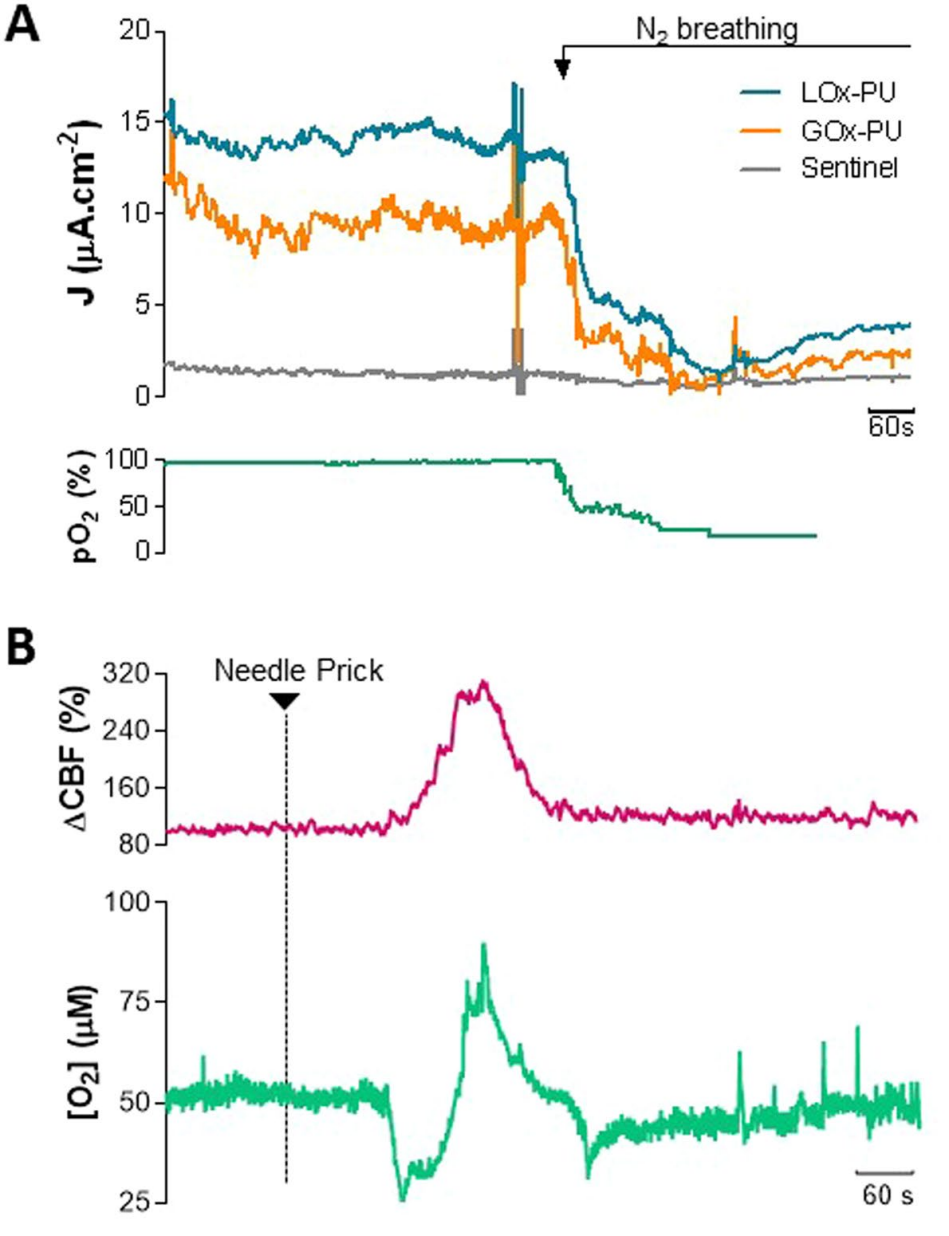
*In vivo* validation of lactate and glucose measurements by the dual biosensor. (**A**) Recording of basal lactate (blue line) and glucose (orange line) concentrations with the LOx-GOx MBA implanted in the cerebral cortex of urethane-anesthetized rats. The detection of lactate and glucose was hampered by anoxic conditions created by forcing the animal to breath pure N_2_ gas (downward arrow). The induced changes in pO_2_ were confirmed systemically by pulse oximetry (green line). (**B**) *In vivo* recording of O_2_ changes (green line) and cerebral blood flow (pink line) in the cerebral cortex of urethane-anesthetized rats in response to SD. SD was induced by a needle prick at the time indicated by the downward arrow.

The capability of the LOx-GOx MBA described herein to measure lactate and glucose concentrations in the brain extracellular space was confirmed *in vivo* by the higher background current of the LOx and GOx-coated sites as compared to the sentinel sites (Fig. 5A). By imposing anoxic conditions, promoted by forcing the animal to breathe pure N_2_ gas, the amperometric currents of active sites were similar to that of the sentinel sites (note that O_2_ is a co-substrate for the LOx and GOx). This supports that the current recorded by LOx and GOx-coated sites results from lactate and glucose oxidation, respectively, with minimal chemical interferences.

**Figure 6:**
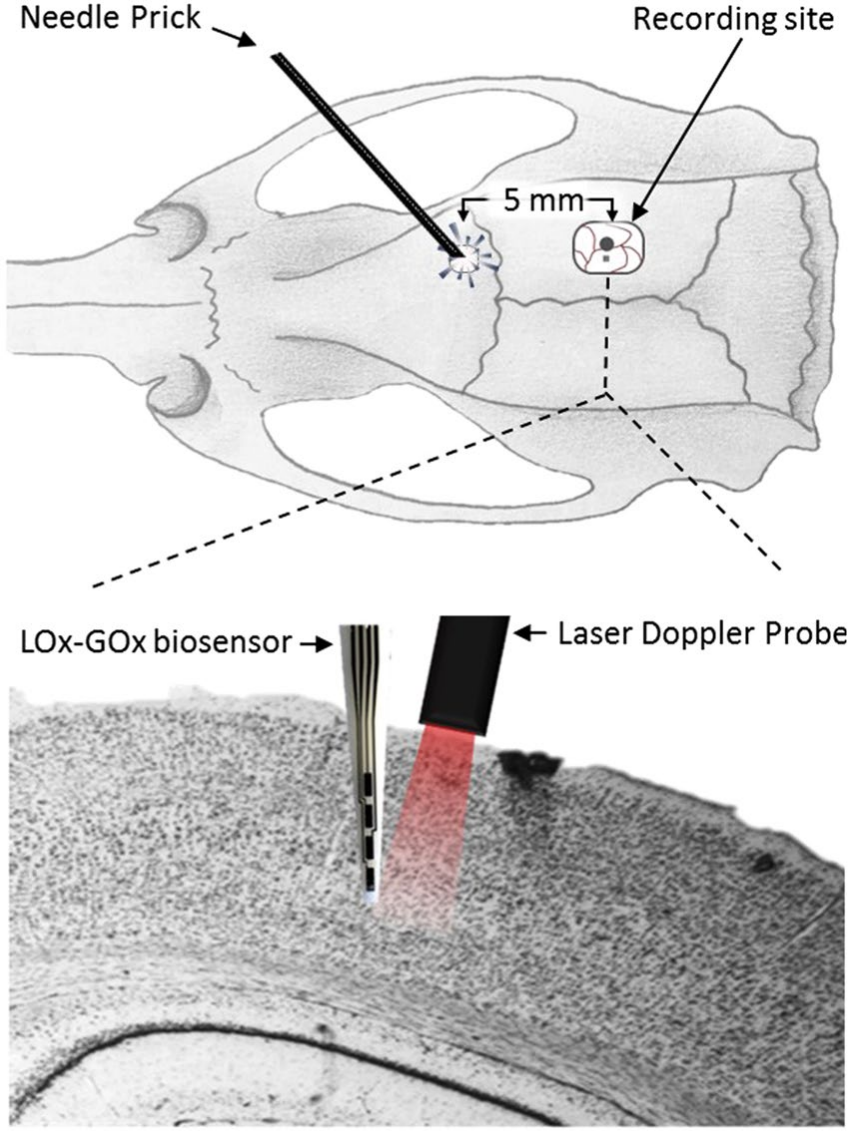
Schematic representation of the *in vivo* setup used to simultaneous measure cerebral blood flow, by laser Doppler Flowmetry, and neurometabolic and electrophysiological changes, by the LOx-GOx MBA, in response to SD depolarization induced by the needle prick of cortical surface.

To define the potential interference of O_2_ fluctuations on the lactate and glucose measurements by LOx-GOx MBA during SD, we further measured the changes in O_2_ concentration in the extracellular space of the rat brain with a sentinel site polarized at a negative potential (−0.6 V vs Ag/AgCl)^18^. The LOx-GOx MBA was inserted in the medial parietal association cortex along with a laser Doppler probe to monitor cortical CBF. SD events were mechanically induced by a needle prick 5 mm away from the recording site as seen in Fig. 6. Continuous monitoring of physiological parameters revealed spontaneous fluctuations of heart and respiration rate and pulse oximetry within a physiological range, without correlation to SD events. The CBF was measured in close proximity of the LOx-GOx MBA to confirm the occurrence of the SD episode. Hyperemia typically starts *ca*. 15 s after the DC shift onset, reaching a maximum after complete repolarization^4^. We observed the hyperemic wave of propagating at 2–5 mm/min characterized by a robust increase in CBF (>100%) that lasted 2 min (Fig. 5B). Within the same time course, we observed a significant change in O_2_ associated with the hyperemic response characterized by an initial significant decrease to 25 µM and a later transient increase to 80 µM (Fig. 5B).

### Measurement of neurometabolic and electrophysiological changes during cortical spreading depolarization

**Figure 7:**
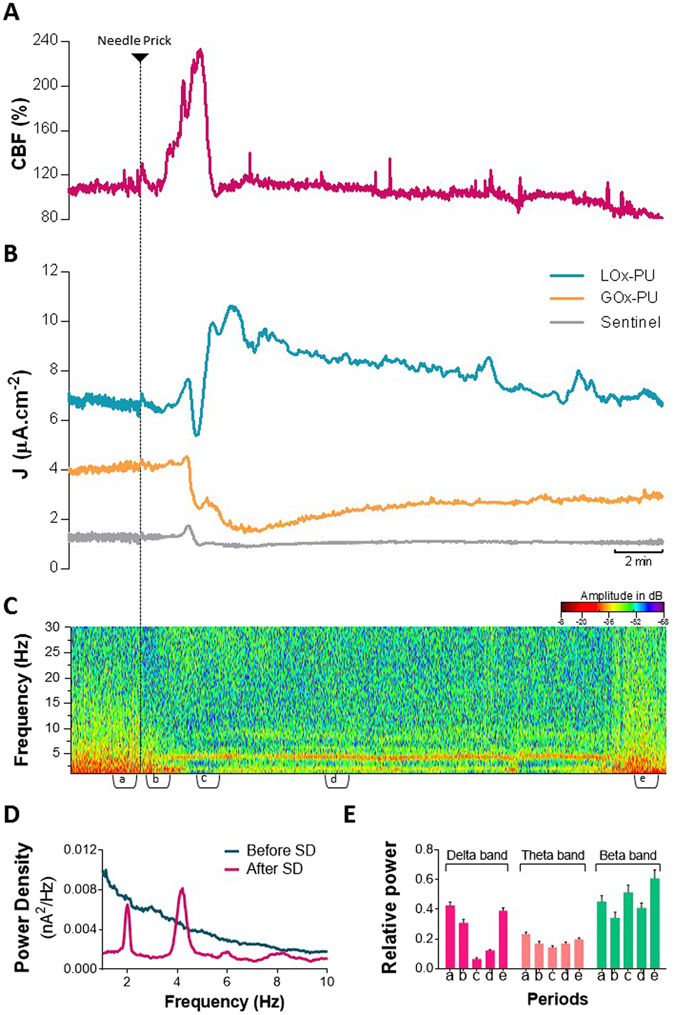
Neurometabolic changes and LFP-related information during cortical SD addressed by (**A**) CBF recording, (**B**) low frequency component (<1 Hz) of the amperometric signals recorded by the LOx-GOx MBA and (**C**) power spectrogram of the higher frequency component (>1 Hz) of the amperometric signal. (**D**) Average power density of the amperometric signals during the 60 s period recorded before (epoch *a*) and after the SD induction (epoch *c*). (**E**) Average relative power of delta (1–3 Hz), theta (3–6 Hz) and beta (14–30 Hz) bands in five different periods (60 s) associated with the SD event: *a* – control condition, *b* – after needle prick, *c* – during hyperemic response, *d* – after hyphemia, *e* – recovered condition. The events are marked in the bottom of the power spectrogram (**C**).

The amperometric recordings with LOx-GOx MBA were performed at high frequency (100 Hz) with the aim of simultaneously measuring changes in lactate and glucose concentrations and LFP-related currents. Figure 7 shows a representative recording of CBF, the low frequency component (<1 Hz) of the amperometric signal recorded by the LOx-PU, GOx-PU and sentinel sites of the LOx-GOx MBA, and the spectral analysis of the higher frequency component (>1 Hz) of the same amperometric signal. The increase in CBF associated with the SD wave occurred along with multiphasic fluctuations in the extracellular concentration of both lactate and glucose. Lactate concentration initially decreased slightly with a minimum coincident with the time of maximal CBF. It then steeply increased to reach a maximum concentration within 4 minutes of SD induction. On average, lactate increased 66% from a basal concentration of 0.83 ± 0.22 mM (n = 3). The basal levels were restored after 20 minutes. Accompanying such changes, we observed a dramatic decline of glucose concentration that reached a minimal value within 5 minutes of SD induction. On average, glucose decreased 51% from a basal concentration of 1.56 ± 0.47 mM (n = 3). This decrease was then followed by a slower and typically incomplete recovery 20 minutes after SD induction. During the recovery period spontaneous lactate oscillations were observed, with no correlation with glucose changes. The sentinel site recorded only a negligible current change. The matched drop in glucose and rise in lactate, resulted in a transient increase of the lactate/glucose ratio of over 300% increase (from an average value of 0.7 prior to SD).

The spectral analysis of LFP-related currents retrieved from the high-frequency component of the amperometric signals revealed relevant alterations in the power spectrum in close relation with the metabolic changes elicited by SD (Fig. 7C). We observed that the SD wave was accompanied by a synchronization of the slow wave activity in delta and theta oscillations reflected by two prominent power bands, respectively, at 2 and 4 Hz (see Fig. 7C and D). The synchronization persisted for *ca.* 20 min and was associated with a decrease in the relative power density of that frequency range (Fig. 7E). Coincident with the hemodynamic response, we observed a significant decrease in the power density in the delta range (1–3 Hz) that lasted *ca.* 1 minute.

## Discussion

In this work we present a multimodal approach, encompassing metabolic, electric and hemodynamic measurements, tailored to study spreading depolarization events. For that purpose we developed, characterized and validated a dual microbiosensor array for lactate and glucose measurements in rat brain tissue with high temporal, spatial and chemical resolution. The detailed *in vitro* characterization revealed that the dual glucose and lactate oxidase- microbiosensor array, based on an efficient glutaraldehyde immobilization procedure following polyurethane coating, exhibit suitable analytical performance towards measurements in brain tissue, in particular a good linear range, high sensitivity and selectivity. In particular the sensitivity and linear range are comparable with data previously reported for planar microbiosensors^30, 31^ and Pt-wire-based microbionsensors^32^ similarly modified with phenylenediamines and polyurethane. The polyurethane membrane is required to extend the linear range, which is critical for lactate measurement considering the low apparent K_M_ of LOx^33^. Coherently, polyurethane promoted a significant increase in K_M_ both for lactate (from 1.4 ± 0.2 mM to 22 ± 6 mM, unpublished) and glucose (from 6.6 ± 0.2 mM^29^ to 80 ± 14 mM), in accordance to data reported by others^16, 30, 32^. This effect is consequence of the diffusion limitation of analyte *versus* O_2_ imposed by the polyurethane layer and unlikely due to an actual change in enzyme kinetics. Overall, the LOx-GOx-MBA array exhibited an appropriate dynamic range to measure large changes in lactate and glucose concentration in brain extracellular space. This expanded linear range was obtained at the expense of a decrease in sensitivity and a slower response time as expected due to the diffusion barrier imposed by the polyurethane membrane, yet it is in a range suitable for the detection of neurometabolic events on a second-by-second time-scale. A much lower dynamic range was reported for Pt/Ir wire-based biosensors used for the same purpose, which indeed is unarticulatable with the magnitude of the glucose and lactate changes reported^5^. The use of polyurethane offers an additional advantage related to a reduction of oxygen dependency obtained by the reduction in the diffusion of the metabolic substrates to the enzymatic matrix relative to that of O_2_^15, 31^. This is of major relevance considering the evidence for relevant changes in pO_2_ accompanying SD events^34^. Coherently, LOx-GOx-MBA array revealed minimal oxygen-dependencies in lactate and glucose measurements. Data support that while increases above physiological concentrations of O_2_ (30–50 µM)^35, 36^ unlikely influence lactate and glucose measurements, decreasing O_2_ below 5 µM could interfere with the amperometric current measurements. The amplitude of O_2_ changes observed *in vivo* in response to the same experimental paradigm used to evaluate the metabolic and electrophysiological changes during SD, supports that the changes in O_2_ concentrations in the brain extracellular space are unlikely to impact lactate and glucose measurements by the LOx-GOx MBA during SD. This further supports the suitability of this array to monitor metabolic changes during SD. Furthermore, lactate and glucose measurements by LOx-GOx-MBA array also revealed minimal dependency of temperature and pH. This is of major relevance, as while these experimental parameters are kept constant in *in vitro* studies, significant changes can occur *in vivo* with impact on the estimation of lactate and glucose concentrations. For instance, there is evidence for pH decreases (~0.4 pH unit)^9^ and temperature oscillations^37^ during SD. Importantly, the magnitude of changes induced by this variables (within a reasonable window of variation) was minimal and in the same extension for both lactate and glucose, thus unlikely to significantly impact on our measurements. In situations expected to cause more dramatic changes in O_2_, pH and temperature, the magnitude of the changes in these parameters should be determined and used to correct the measurements of lactate and glucose for those conditions. In this context, we must caveat that our experimental paradigm of SD was associated with a hemodynamic response mainly characterized by a dominant hyperemic response, which is typically observed in healthy and well perfused brain tissue. In injured brains, depending on the type of injury and its severity, SD events may be instead associated to an inverse neurovascular coupling^38–40^.

By using amperometric recordings *in vivo* with the same LOx-GOx-MBA array we successfully measured the metabolic signature of SD simultaneously with pronounced changes in the spectral profile of LFP-related currents, in correlation with CBF changes induced by a needle prick model of traumatic brain injury. In spite of some differences in the magnitude of the changes and the temporal profile, the variations in lactate and glucose here reported followed the known metabolic signature of SD, globally characterized by a fall in glucose extracellular concentration and an increase in lactate^5–9^. The significant drop in glucose concentration occurs due to glycolysis stimulation in response to a massive increase in oxidative phosphorylation to generate the ATP required to restore the ionic gradients lost during the SD wave. In spite of the increase in glucose supply by the hyperemic response, the energetic demands are such that this input is insufficient to meet demand. The accumulation of lactate in the brain extracellular space, while accepted, is more puzzling to understand, particularly under conditions of available O_2_^41^_._ The observed relative changes in lactate are in agreement with those reported using rsMD^6–8^, but the higher temporal resolution of the LOx-GOx-MBA array revealed multiphasic and more complex fluctuations in lactate extracellular concentration. In spite of different amplitudes, the biphasic change in lactate associated with SD was previously detected using electrochemical biosensors directly implanted in the brain parenchyma^5^. In turn, the magnitude of the changes here reported for glucose were higher than those revealed by rsMD^6–8^. The larger changes in metabolites detected using biosensors are suggested to occur due to a low-pass filtering of the fast neurometabolic changes in rsMD^5^. However, it should be stressed that these comparisons are of relative value considering the experimental differences, both the method for metabolites monitoring, but also in the anesthetic used, the stimuli for SD induction and the strain and age of the rats studied. The matched drop in glucose and rise in lactate, resulted in a transient increase of the lactate/glucose ratio which is suggested to be a biomarker of poor outcome in TBI and other neuropathological conditions^11, 42, 43^.

A major observation in this work was the marked alterations in the spectral profile of LFP-related currents accompanying the metabolic changes during and after SD. Most significantly, we observed a synchronization of the slow wave activity in the delta and theta range accompanying the period of metabolic disturbance. This time window is within the range of the period of the depression of spontaneous and evoked synaptic activity following SD^4^. Interestingly, alterations in delta and theta activity were also observed in the EEG of patients during attacks of migraine with prolonged aura^44^, a condition associated to SD events^45^. Furthermore, we observed a significant decrease in the power of the delta band during hemodynamic response. We speculate that this decrease is a fingerprint of the DC negative shift and may be used as surrogate marker for the SD wave. In agreement, it was shown that >80% of SDs recorded from EcoG in brain trauma manifest as depressions of high-amplitude delta activity in EEG recording^24^. This evidence supports the possibility of identifying SD waves by spectral analysis without recording DC potential as suggested by others^46^. Yet, simultaneous measurements of DC and amperometric recordings required to validate this hypothesis. In the recent years, emerging evidence has been focused on the changes in EEG profile induced by SD, particularly in the context of TBI, but a clear picture is still to be defined^22^.

While our observations focused on the response to a first SD triggered event, we do not exclude the occurrence of prior SD events considering that craniotomy and microelectrode insertion are reported to potentially cause SD^47, 48^. Indeed, a monophasic CBF increase without detectable initial hypoperfusion or relevant post-oligemia similar to that reported here is usually considered a fingerprint of subsequent SD events^4^. This is relevant considering that in addition to hemodynamic response, the metabolic changes and the spectral profile may vary with the number of SD events^13, 49^.

Overall, in this work we successfully demonstrated the innovative capability to monitor metabolic alterations and ongoing network electrical activity with a unique spatial and temporal resolution using a single array. This multimodal approach is a valuable tool with the potential to be used in basic research to expand the understanding of SD events and their impact on neuronal function and, in the future, to be adapted for clinical setting translation assisting in prognosis and definition of personalized therapeutic strategies^40^. Finally, we anticipate the application of this multimodal approach to not be limited to the SD context. Indeed, in the brain, this approach, coupled to pharmacology, can be useful to provide relevant insights that help to deep the knowledge of the neurovascular and neurometabolic coupling both in physiological and in pathological conditions^30, 38^. Another potential application is cancer, in which LOx-GOx-MBA can pave the way to novel experimental approaches to evaluate metabolic profiling in tumor microenvironment^32^.

## Methods

### Chemicals and solutions

Lactate Oxidase (LOx) (EC 1.13.12.4) from *Pediococcus sp* in powder form, Glucose Oxidase (GOx) (EC 1.1.3.4, Type VII) from *Aspergillus niger* in powder form, *meta*-phenylenediamine (*m*-PD), polyurethane, tetrahydrofuran, sodium lactate, D-(+)-Glucose, ascorbic acid, dopamine, hydrogen peroxide, bovine serum albumin (BSA), glutaraldehyde solution (25%) and urethane were obtained from Sigma-Aldrich. All other reagents were purchased from Merck, unless otherwise specified. All solutions were prepared in bi-deionized MilliQ water with resistivity ≥18 MΩ cm (Millipore Corporation, USA). The electrolyte for *in vitro* analytical evaluation of microbiosensors was phosphate buffer saline (PBS) 0.05 M (pH 7.4) with the following composition (mM): 100 NaCl, 10 NaH_2_PO_4_ and 40 Na_2_HPO_4_. For the pH dependency studies the pH of PBS was priorly adjusted from 5.5 to 8.5 using HCl or NaOH solutions. Stock solutions of glucose (1 M) were allowed to equilibrate the β/α anomeric ratio for 24 h before use.

### Preparation and modification of Lactate-Glucose microbiosensor arrays

Ceramic-based MEAs were obtained from Center for Microelectrode Technology, University of Kentucky, USA. The R1 design configuration containing 4 sites (50 × 150 µm^2^) in-line separated by 50 µm was used as the microelectrode platform for construction of the dual microbiosensor array (Fig. 1). Lactate (LOx) and Glucose oxidase (GOx) were immobilized differentially onto the Pt sites using a coating procedure essentially as described^29, 31^. The cocktail solutions of enzymes were differentially applied to the sites surface using a microsyringe mounted on micromanipulator under a stereomicroscope using the following scheme: site 1 - LOx (3 mg/mL), BSA (1%) and glutaraldehyde (0.125%) in water and site 3 – GOx (1 mg/mL), BSA (1%) and glutaraldehyde (0.125%) in water. The remaining sites (2 and 4) were coated with the inactive protein matrix solution containing BSA (1%) and glutaraldehyde (0.125%) using the same procedure (Fig. 1). These recording sites should be insensitive to both lactate and glucose and as such act as sentinel or null sites. The dual microbiosensors were stored dry in the dark and protected from dust, at room temperature, for at least three days to allow for curing and stabilization of the active and non-active matrices. Then, a polyurethane membrane was deposited by dip coating in a solution of polyurethane 2% dissolved in a mixture of tetrahydrofluran (98%) and dimethylformamide (2%) and allowed to dry for 24 hours. To minimize access of undesirable electrochemically active compounds to the Pt recording sites, the MEA-based GOx biosensors were modified, before use, with an exclusion layer of *m*-PD. Briefly, the *m*-PD solution (10 mM) was freshly prepared in deoxygenated PBS and electropolymerized at the MEA Pt surface using the FAST16mkIII high-speed electrochemical system (Quanteon, Nicholasville, KY, USA) by cyclic voltammetry between +0.25 and +0.75 V *vs* an Ag/AgCl reference electrode (RE-5, BAS Inc., USA) at a scan rate of 50 mV/s during 20 minutes. For some *in vitro* studies, S2 MEA design configuration was used as a microelectrode platform for biosensor construction, without the use of sentinel sites.

### *In vitro* evaluation and characterization

The *in vitro* evaluation of the LOx-GOx MBA for measurement of lactate and glucose was performed by amperometry at +0.7 V *vs* Ag/AgCl (RE-5, BAS Inc., USA) using the FAST16mkIII high-speed electrochemical system in a two-electrode configuration mode. The experiments were carried out in 40 mL PBS 0.05 M at 37 °C under gentle stirring after a 30 minute period of current stabilization. Analytical and kinetic parameters were determined after baseline stabilization by adding aliquots of the stock lactate or glucose solution to obtain final concentrations in the range of 0.05 to 20 mM and 0.125 to 60 mM, respectively. The sensitivity to lactate and glucose, selectivity against major interferents and the sensitivity to the reporter molecule were determined by three additions of lactate and glucose (0.5 mM) in the presence of ascorbic acid 0.5 mM, followed by DA 10 µM and H_2_O_2_ 10 µM.

To evaluate the dependency of LOx-GOx MBAs for O_2_, temperature and pH, the responses to glucose and lactate (1 mM) were measured under variable O_2_ concentrations, temperature and pH, essentially as previously described^29^.

### Surgical procedures and *in vivo* experiments

All the procedures used in this study were performed in accordance with the European Union Council Directive for the Care and Use of Laboratory animals, 2010/63/EU and were approved by the local ethics committee of the animal house facilities of the Center for Neurosciences and Cell Biology (ORBEA - *Órgão Responsável pelo Bem-Estar Animal*). *In vivo* studies were carried out on adult male Wistar rats (10 weeks) maintained in controlled environmental conditions: temperature of 22–24 °C, relative humidity of 45–65%, 15 air exchanges per hour and a 12:12 light/dark cycle. Animals were housed in filter-topped type III Makrolon cages on an individually ventilated caging system (VentiRack Bioscreen™). Rats were fed with a standard rat chow diet (4RF21-GLP Mucedola, SRL, Settimo Milanese, Italy) and were provided with chlorinated water, both *ad libitum*.

Rats were anesthetized with urethane (1.25 g/kg, i.p.) and a stereotaxic surgery was performed as previously described^29^. Animals breathed spontaneously and their body temperature was maintained at 37 °C using a heated pad coupled to a Gaymar Heating Pump (Braintree Scientific, Inc., USA). Basic physiological parameters (blood O_2_ saturation, heart rate and breath rate) were continuously monitored during the experiment using a pulse oximeter system (MouseOx^®^, Starr life sciences, Oakmont, PA, USA). After exposing the skull, two craniotomies were performed: a primary posterior craniotomy overlying the parietal cortex (2.5 to 4.5 mm posterior and 1.5 to 3.5 mm lateral to bregma) for measurements and a secondary craniotomy (~1 mm^2^, 5 mm posterior to the primary craniotomy) for SD induction. The meninges were removed from the brain surface prior to the insertion of the pre-calibrated LOx-GOx MBA into the rat cerebral cortex according to coordinates calculated from *bregma* based on the rat brain atlas of Paxinos and Watson^50^: 3.6 mm posterior, 2.0 mm lateral and 1.8 mm ventral to bregma. The LOx-GOx MBA was positioned with the site’s surface in posterior orientation. An Ag/AgCl reference electrode prepared from a silver wire (200 µm diameter) was placed underneath the retracted skin and kept moistened with saline. Electrochemical recordings were performed using the FAST16mkIII high-speed electrochemical system (Quanteon, Nicholasville, KY, USA) applying a constant potential (+0.7 V vs Ag/AgCl) and using a 100 Hz acquisition rate. Cerebral blood flow was simultaneous and continuously measured by laser Doppler flowmetry (Periflux System 5000, Perimed AB, Järfälla, Sweden). The LDF probe (780 nm wavelength, 250 µm fiber separation) was placed on the cortical surface in the immediacy of the MBA. Calibration of the probe was performed routinely according to manufacturer recommendations to equalize the perfusion values among the various recordings. The time constant was set to 0.03 s and the signal-processing unit used a bandwidth of 32 Hz. After a 1 hour stabilization period, SD was induced 5 mm away from the recording site by pricking the upper cortical layers with the tip of a 26-gauge needle as previously described^6, 8^. As a control experiment, anoxia was induced by forcing the animal to breath pure N_2_ gas until cardiac arrest was achieved.

### Data Analysis

*In vitro* data analysis was performed using GraphPad Prism 5.0. For the kinetic analyses, data were fitted to a Michaelis-Menten type equation and the maximum steady-state current response normalized by the geometric area of the active Pt site (J_max_) and apparent Michaelis-Menten constants (K_M_) for lactate and glucose were determined. The selectivity ratio, on a molar basis, against ascorbate was calculated from the recording of the enzyme-coated sites as the ratio of the sensitivities for lactate or glucose and ascorbate. The crosstalk between sites is defined by the *in vitro* response detected in an inactive site (sentinels) following the addition of glucose or lactate The limit of detection (LOD) was defined as the concentration that corresponds to a signal-to-noise ratio of 3. The response time (t_90%_ - t_10%_) of the microbiosensors was determined by fitting the data to a Boltzmann sigmoid function and calculating the time interval between 10% and 90% of the maximal response to lactate or glucose (1 mM). For the O_2_ dependency studies, the apparent Michaelis-Menten constant for O_2_ (K_M_(O_2_)) was determined under fixed lactate and glucose concentrations by fitting the normalized data to a Michaelis-Menten type equation. Values are given as the mean ± standard error of the mean (SEM).

The signal processing of *in vivo* recordings was performed using OriginPro2016. The amperometric recordings were low passed filtered at 1 Hz to extract the electrochemical component of the oxidation currents associated to lactate, glucose and potential interferents. Power spectrograms were constructed using the short-time Fourier transform of the unprocessed amperometric signals. The power density relative to the periods before and after SD was calculated by averaging the Fast Fourier transform of 60 s epochs. The relative powers of delta (1–3 Hz), theta (3–6 Hz) and beta (14–30 Hz) bands were calculated by ratio of the power of the band and total power.
